# Synergy of NUP98-HOXA10 Fusion Gene and NrasG12D Mutation Preserves the Stemness of Hematopoietic Stem Cells on Culture Condition

**DOI:** 10.3390/cells8090951

**Published:** 2019-08-22

**Authors:** Yong Dong, Chengxiang Xia, Qitong Weng, Tongjie Wang, Fangxiao Hu, Kaitao Wang, Xiaofei Liu, Yang Geng, Lijuan Liu, Hongling Wu, Juan Du

**Affiliations:** 1 Key Laboratory of Regenerative Biology of Chinese Academy of Sciences, Guangzhou Institutes of Biomedicine and Health, Guangzhou 510530, China; 2 Guangdong Provincial Key Laboratory of Stem Cell and Regenerative Medicine, Guangzhou Institutes of Biomedicine and Health, Guangzhou 510530, China; 3 University of Chinese Academy of Sciences, Beijing 100049, China; 4 Joint School of Life Sciences, Guangzhou Medical University, Guangzhou 510530, China; 5 Guangzhou Regenerative Medicine and Health Guangdong Laboratory, Guangzhou 510005, China

**Keywords:** NUP98-HOXA10HD, NrasG12D, hematopoietic stem cells

## Abstract

Natural hematopoietic stem cells (HSC) are susceptible and tend to lose stemness, differentiate, or die on culture condition in vitro, which adds technical challenge for maintaining *bona fide* HSC-like cells, if ever generated, in protocol screening from pluripotent stem cells. It remains largely unknown whether gene-editing of endogenous genes can genetically empower HSC to endure the culture stress and preserve stemness. In this study, we revealed that both NUP98-HOXA10HD fusion and endogenous Nras mutation modifications (NrasG12D) promoted the engraftment competitiveness of HSC. Furthermore, the synergy of these two genetic modifications endowed HSC with super competitiveness in vivo. Strikingly, single NAV-HSC successfully maintained its stemness and showed robust multi-lineage engraftments after undergoing the in vitro culture. Mechanistically, NUP98-HOXA10HD fusion and NrasG12D mutation distinctly altered multiple pathways involving the cell cycle, cell division, and DNA replication, and distinctly regulated stemness-related genes including *Hoxa9*, *Prdm16*, *Hoxb4*, *Trim27*, and *Smarcc1* in the context of HSC. Thus, we develop a super-sensitive transgenic model reporting the existence of HSC at the single cell level on culture condition, which could be beneficial for protocol screening of *bona fide* HSC regeneration from pluripotent stem cells in vitro.

## 1. Introduction

Single cell transplantation of fresh wild type hematopoietic stem cells (HSC) can successfully repopulate multi-lineage hematopoiesis in myeloid-ablated mice [1,2,3]. However, the success rates of engraftment remained low in recipients when using 1% donor cell contribution as a readout of successful engraftment. In addition, unlike induced pluripotent stem cells, which can be reported by the OCT4 gene reporter, currently, there is no single gene acting as a reporter that can robustly mark bona fide HSC in vitro. Various functional tests for HSC have been developed [3]. However, it is still necessary to produce genetically enhanced HSC for a more sensitive stemness-readout of HSC in vitro.

Natural HSC tend to lose their stemness, differentiate to lineage offspring, or die on the condition of in vitro culture stress. Although there have been many reports that HSC proliferated in vitro in the presence of certain cytokines, chemical compounds, and feeder cells [4,5,6,7,8], the clinical application of in vitro expanded HSC for disease treatment is still lagging behind. Alternative resource of HSC, such as induced HSC from induced pluripotent stem cell (iPSC), is urgently needed for universal clinical applications. Among various technical challenges, a sensitive reporting system of bona fide HSC is essential for large-scale screening approaches to regenerate induced HSC in vitro from iPSC.

*Hoxb4* has been reported to be the key element in HSC stemness and was used to mediate pluripotent stem cell differentiation toward HSC [9,10]. Since NUP98-HOXA10 was reported to expand HSC more efficiently than HoxB4 and the leukemogenic effect and HSC-expanding effect of NUP98-HOXA10 fusion protein can even be separated by a new artificial fusion form of NUP98-HOXA10HD [8]. Moreover, we and others previously reported that the mutant NrasG12D HSC showed a competitive engraftment advantage [11]. However, whether NUP98-HOXA10HD and NrasG12D represent ideal genetic modification to sensitively report the existence of HSC on a culture condition requires further study.

In this study, we compared the effects of the NrasG12D mutation and the NUP98-HOXA10HD fusion gene on the engraftment competitiveness of HSC and their combinative role in preserving the stemness of HSC after in vitro culture stress. Despite that both the NUP98-HOX10HD fusion gene and the NrasG12D mutation enhance the competitiveness of HSC engraftment, they employ distinct signaling mechanisms and the synergy of these two factors result in super competitiveness in vivo in modified HSC (NAV-HSC). The single NAV-HSC preserved their stemness after a 10-day feeder-free culture in vitro and showed robust multi-lineage engraftments in vivo upon transplantation. Thus, we developed a super-sensitive model reporting the existence of HSC at the single cell resolution, which is beneficial for protocol screening of *bona fide* HSC regeneration from pluripotent stem cells in vitro.

## 2. Results

### 2.1. NUP98-HOXA10HD-Knock-In Mice Show Normal Hematopoiesis with Decreased Hematopoietic Stem and Progenitor Compartment

**Figure 1:**
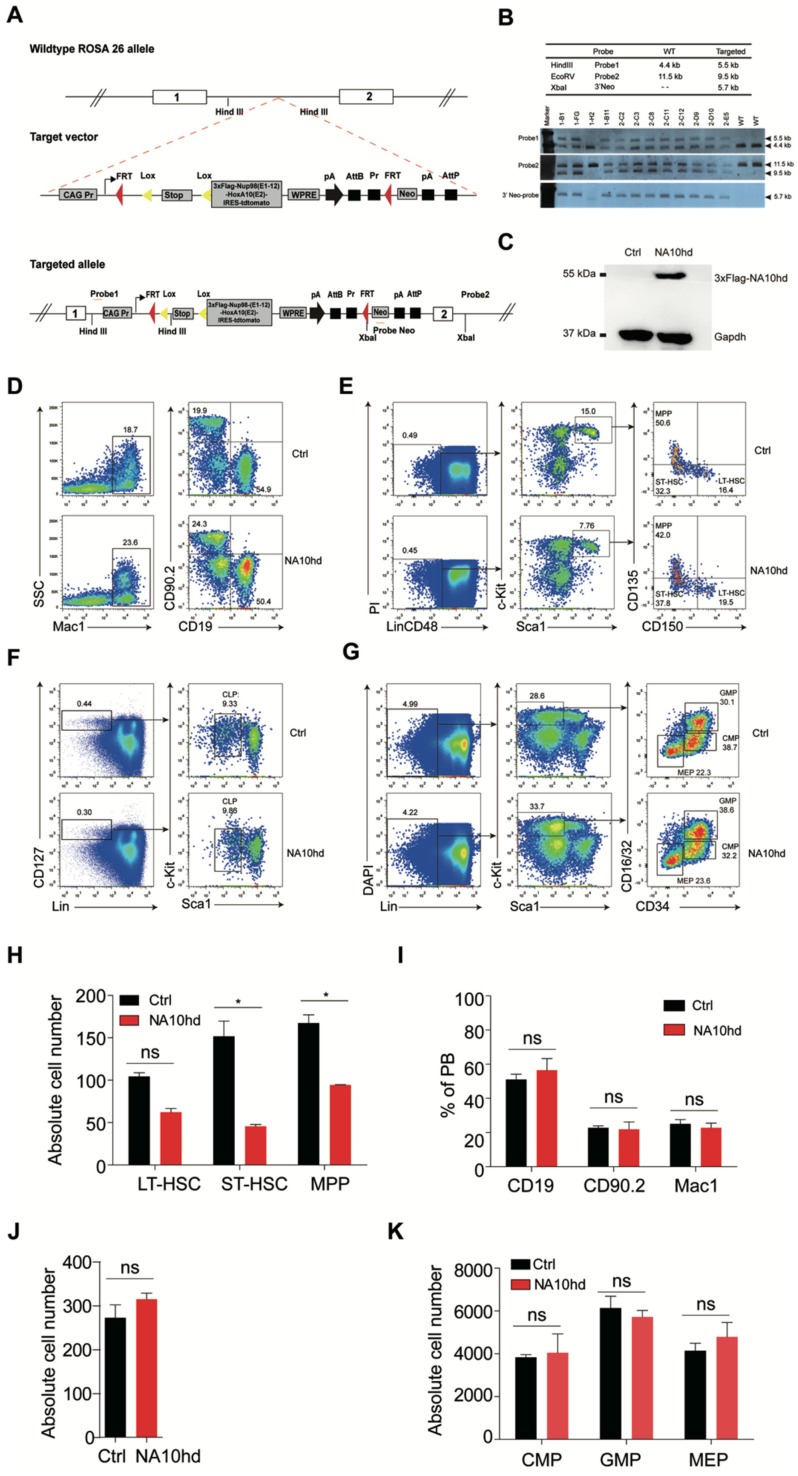
Establishment and analysis of multi-lineage hematopoiesis and hematopoietic progenitors in NA10hd transgenic mice. (**A**) Schematic diagram of mouse embryonic stem cell (ESC) targeting strategy for 3xFlag-NA10hd expression elements in the ROSA26 locus. (**B**) Southern blot analysis of targeted ESC clones. (**C**) Western blot of 3xFlag-NA10hd fusion protein in NA10hd^LSL/+^; Vav-Cre mice. Bone marrow nucleated cells of NA10hd^LSL/+^; Vav-Cre mice or control mice (NA^LSL/+^, Ctrl) were analyzed. (**D**–**G**) Flow cytometric analysis of hematopoietic lineages in peripheral blood and bone marrow. Representative flow plots of hematopoietic lineage analysis in peripheral blood (**D**), hematopoietic stem/progenitor cell (hematopoietic stem cell, HSC/multipotent progenitor, MPP) (**E**), common lymphoid progenitor (CLP) (**F**), and myeloid progenitor (MP) (**G**) in bone marrow of NA10hd and control mice are shown. Data are representative of three independent experiments. (**H**) The absolute cell number of Lin^−^CD48^−^c-Kit^+^Sca1^+^CD135^+^CD150^−^ MPP, Lin^−^CD48^−^c-Kit^+^Sca1^+^CD135^−^CD150^−^ ST-HSC, and Lin^−^CD48^−^c-Kit^+^Sca1^+^CD135^−^CD150^+^ LT-HSC in one million bone marrow cells of NA10hd and control mice was calculated. Lin cocktail includes CD2, CD3, CD4, CD8, B220, Mac1, Gr1, and Ter119. (**I**) The percentages of Mac1^+^ myeloid cells and CD19^+^ B cells and CD90.2^+^ T cells in peripheral blood of NA10hd mice. (**J**–**K**) The absolute number of Lin^−^CD127^+^c-Kit^mid^Sca1^+^CLP (**J**), Lin^−^CD127^−^c-Kit^+^Sca1^+^CD16/32^+^CD34^+^ GMP, Lin^−^CD127^−^c-Kit^+^Sca1^+^CD16/32^mid^CD34^mid^ CMP, and Lin^−^CD12^−^c-Kit^+^Sca1^+^CD16/32^−^CD34^−^ MEP (**K**) in one million bone marrow cells of *NA10hd* and control mice was calculated based on a respective percentage measured by flow cytometric analysis. Data are represented as means ± SD. An unpaired Student’s t-test (two-tailed) was performed. *N* = 3 mice, * *p* < 0.05, ns indicates not significant.

Overexpression of the NUP98-HOXA10HD fusion protein promotes expansion of both mouse and human HSC in vitro [12,13]. In this scenario, we established an NA10hd knock-in transgenic mouse by inserting the NA10hd expression elements into the ROSA26 locus of mouse embryonic stem cells (C57BL/6 background). For easy measurement of NA10hd expression at a protein level, we inserted a 3xFlag sequence at the end of the NA10hd sequence. To report the expression of NA10hd, we added a sequence encoding the Tdtomato fluorescent protein after the internal ribosome entry site (IRES) following the NA10hd sequence (Figure 1A). The expression of NA10hd is locked by a loxp-stop-loxp (LSL) cassette and can be activated in a tissue-specific manner by a Cre line. A Southern blot identified the recombinant ES cells (Figure 1B). NA10hd conditional expression mice (LSL-NA10hd) were generated by blastocyst injection. To express NA10hd in the hematopoietic system, the LSL-NA10hd mice were further crossed to Vav-Cre mice (C57BL/6 background) to produce LSL-NA10hd and Vav-Cre compound mice (NA10hd mice, CD45.2^+^). A Western blot using antibodies and recognizing the 3xFlag confirmed the expression of NA10hd-3xFlag protein in the bone marrow nucleated cells of NA10hd mice (Figure 1C).

To assess the effect of NA10hd on hematopoiesis, we performed flow cytometric analysis on multi-blood lineages in peripheral blood (Figure 1D), hematopoietic stem cells, multi-potential progenitors cells (Figure 1E), and myeloid and lymphoid progenitors (Figure 1F,G), in bone marrow of NA10hd and littermate control mice. The absolute cell number of hematopoietic stem cells and multilineage progenitor cells of NA10hd was mildly less than those of control mice (Figure 1H). However, the absolute number of the mature myeloid or lymphoid cells in the peripheral blood (Figure 1I), lymphoid progenitor cells (Figure 1J), and myeloid progenitors (Figure 1K) in the bone marrow was comparable between NA10hd and the control. Analysis results from 12-month-old aged NA10hd mice were largely similar, despite the number of long-term HSC and lymphoid progenitors being significantly less than the littermate control (Figure S1). Taken together, NA10hd-expresssing mice generally show normal hematopoieisis despite having a decreased number of hematopoietic stem and progenitor cells.

### 2.2. NUP98-HOXA10HD Expression Confers Competitive Transplantation Advantage on HSC

**Figure 2:**
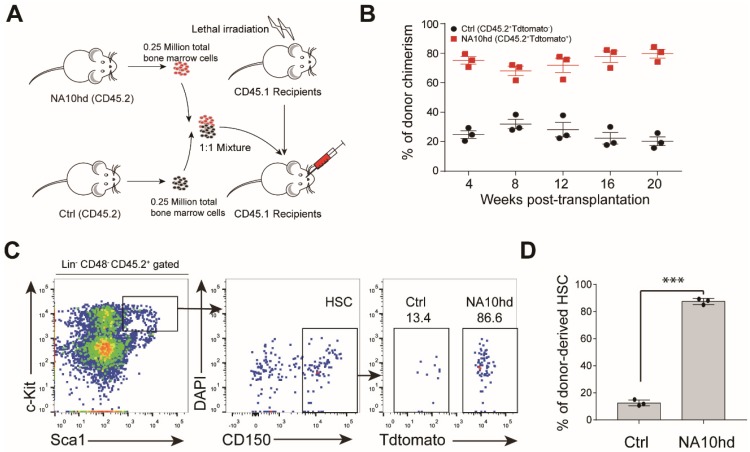
Hematopoietic stem cells expressing NA10hd protein demonstrates a competitive engraft advantage. (**A**) Diagram of the competitive transplantation assay of NA10hd and the littermate control (NA10hd^LSL/+^, CD45.2) bone marrow hematopoietic stem cells. NA10hd and control bone marrow nucleated cells were transplanted into lethally irradiated (2 × 5.0 Gy) recipients (CD45.1) at a ratio of 1:1, and the percentage of donor cells and donor-derived specific lineage within donor-derived cells in peripheral blood were analyzed regularly after transplantation. (**B**) Donor chimerism in peripheral blood. (**C**) Flow cytometric analysis of donor-derived (NA10hd or control) hematopoietic stem cell (HSC) proportion in bone marrow of recipients 20 weeks post transplantation. Data are representative of two independent experiments. (**D**) Statistical analysis of *NA10h*d or control donor-derived HSC in recipient bone marrow 20 weeks post-transplantation. Data are represented as means ± SD. Unpaired Student’s t-test (two-tailed) was performed. Three mice, *** *p* < 0.001.

To further assess the impact of NA10hd on engraftment and lineage contribution, we retro-orbitally injected 2.5 × 10^5^ total bone marrow cells from NA10hd mice into lethal irratidation (2 × 5.0 Gy) treated mice (CD45.1) together with an equal number of bone marrow competitor cells from littermate control (NA10hd^LSL/+^) mice (CD45.2) (Figure 2A). These recipients were bled every four weeks retro-orbitally to evaluate the multi-lineage reconstitution capacity of transplanted cells. The recipients showed a signifcantly higher proportion of NA10hd-cell chimersim (>70%) in peripheral blood (Figure 2B) starting from week 4 until week 20. We then asked whether dominance of NA10hd in periheral blood is a result of dominance at the HSC level. Chimerism of the HSC compartment were examined in the bone marrow of recipient mice 20 weeks after transplantion. As expected, NA10hd-HSC composed of more than 80% of the HSC compartment, which was significantly higher than the control HSC (*p* < 0.001) (Figure 2C,D). These results show that NA10hd confered a competitive advantage on hematopoietic stem cells (HSC) in the transplantation setting.

### 2.3. Synergistic Effect of NUP98-HOXA10HD and NrasG12D on the Competitiveness of HSC In Vivo

**Figure 3:**
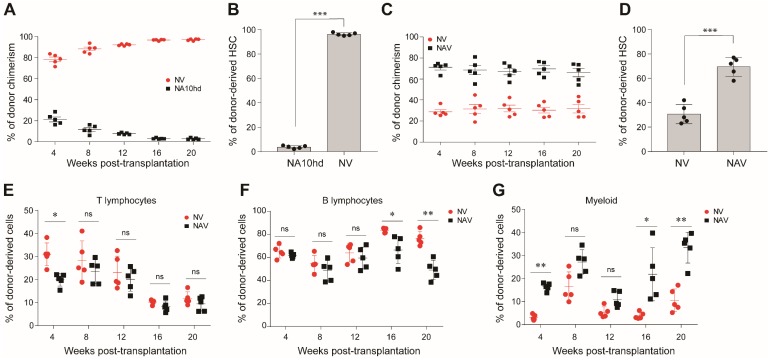
Comparison of engraftment competitiveness of NA10hd and NrasG12D^LSL/+^; Vav1-Cre (NV) hematopoietic stem cells. (**A**) Donor-derived cell chimerism in peripheral blood. NV and NA10hd bone marrow nucleated cells were transplanted into lethally irradiated (2 × 5.0 Gy) recipients (CD45.1) at a ratio of 1:1, and the percentage of donor cells in peripheral blood was analyzed regularly after transplantation. (**B**) The proportion of NA10hd or NV HSC in recipient bone marrow was measured by flow cytometric analysis 20 weeks post-transplantation. (**C**) Donor-derived cell chimerism in peripheral blood. NAV and NV bone marrow were transplanted into lethally irradiated (2 × 5.0 Gy) recipients (CD45.1) at a ratio of 1:1, and the donor cells in peripheral blood was analyzed regularly after transplantation. (**D**) The proportion of NV or NAV HSC were measured by flow cytometric analysis 20 weeks post-transplantation. (**E**–**G**) Donor-derived percentages of T lymphocytes (**E**), B lymphocytes (**F**), and myeloid cells (**G**) in peripheral blood were analyzed regualrly after transplantation. Data are represented as means ± SD. Unpaired Student’s t-test (two-tailed) was performed. *N* = 5 mice. * *p* < 0.05, ** *p* < 0.01, *** *p* < 0.001, ns indicates no significant. NrasG12D and NUP98-HOXA10HD enhance the competitiveness of HSC via distinct signaling mechanisms.

The NrasG12D oncogenic gene confers a competitive advantage on HSC [14,15]. To compare the effects of NrasG12D mutation (NV) and NA10hd on HSC competitiveness, we injected 2.5 × 10^5^ NrasG12D-expressing total bone marrow cells (NV) together with an equal number of NA10hd cell counterparts into lethal irratidation (2 × 5.0 Gy) treated mice (CD45.1). Donor-cell chimerism was analyzed every four weeks post-transplantation. NV cells dominated the peripheral blood from week 4 and contributed significantly higher (>95%) than NA10hd cells (<5%) in the peripheral blood of recipients 20 weeks post-transplantation (Figure 3A). Consistently, we observed that NV-HSC (>90%) rather than *NA10h*d-HSC (<10%) dominantly occupied the HSC compartment in bone marrow of recipients 20 weeks post-transplantation (*p* < 0.001) (Figure 3B). Thus, our results indicate that NV confers stronger competitiveness on HSC than *NA10hd* in the transplantation setting.

Next, we investigated the accumulative effect of NA10hd and NV on HSC competitiveness. The NV and NA10hd strain were bred together with Vav-Cre mice to obtain compound (NAV) mice, which simultaneously express NUP98-HOXA10HD and NrasG12D proteins in the circulatory system. We subsequently transplanted 2.5 × 10^5^ bone marrow nuleated cells from NAV mice, together with an equal number of bone marrow competiter cells from NV mice, into lethally irratidated (2 × 5.0 Gy) recipients (CD45.1). The conribution of NAV-derived cells in peripheral blood was stable and higher than NV and exceeded 60% post transplantation (Figure 3C). In bone marrow, the NAV-derived HSC were also dominant (*p* < 0.001) (Figure 3D). NV-derived T cells were significantly higher than NAV at four weeks but became comparable to NAV thereafter. NV-drived B cells were much higher than NAV at week 16 and week 20, while NV-derived myeloid cells were significantly lower than NAV at week 4, week 16, and week 20. Collectively, both the NrasG12D mutation and the NA10hd fusion gene conferred competitiveness on HSC, and a combination of these two factors produced the strongest accumulative effects on HSC engraftment. Thus, the order of engraftment competitiveness is as follows: NAV-HSC > NV HSC > NA10hd HSC > WT HSC.

### 2.4. NUP98-HOXA10HD and NrasG12D Enhance the Competitiveness of HSC via Distinct Signaling Mechanisms

**Figure 4:**
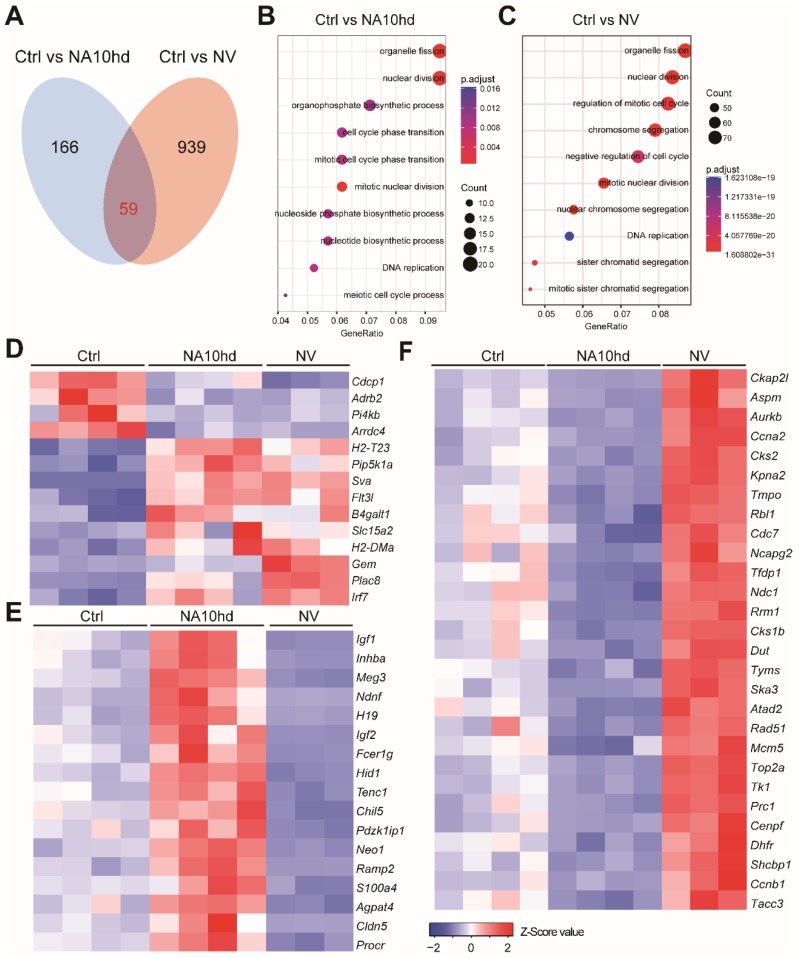
Gene expression pattern in HSC altered by NA10hd and NV expression. For each RNA-Seq sample, one thousand HSC (Lin^−^c-Kit^+^Sca1^+^CD135^−^CD150^+^) from NA10hd, NV, or littermate control mice were sorted as input cells. The raw reads (fastq files) were aligned to NCBIM37 mouse genome by hisat2 software. Gene expression value (raw counts) was extracted using featureCounts. DESeq2 was used to normalize the gene expression matrix and find out the differential expression genes (DEGs). (**A**) Overlap DEGs (p.adj < 0.05, fold change > 1.5) in NA10hd-HSC and NV-HSC compared with control (Ctrl) HSC. (**B**) GO enrichment (EGO) analysis of differential expression genes (DEGs) in NA10hd-HSC. The EGO analysis was performed by a clusterProfiler R package. (**C**) GO enrichment (EGO) analysis of DEGs in NV-HSC. (**D**) Heatmaps of overlapping DEGs with a similar expression alteration trend in NA10hd-HSC and NV-HSC. (**E**) Heatmaps of DEGs that were up-regulated in NA10hd but down-regulated in NV-HSC. (**F**) Heatmaps of DEGs that were down-regulated in NA10hd but up-regulated in NV-HSC. Heatmaps were plotted by gplots (heatmap.2). Columns represent the indicated biological replicates of each population (**D**–**F**).

To investigate the molecular mechanism by which NV and NA10hd enhanced the competitiveness of HSC, we sorted Lin (CD2, CD3, CD4, CD8, B220, Gr1, Mac1, Ter119)^−^CD48^−^c-Kit^+^Sca1^+^CD135^−^CD150^+^ (Figure S2) HSC from NV, NA10hd, and wild-type mice for transcriptome analysis. Since one thousand cell input for RNA-Seq produced reads uniformly covers all transcripts [16], we sorted one-thousand HSC aliquots for RNA-Seq analysis. Differential gene expression analysis identified 225 differential expression genes (DEGs) in NA10hd HSC (Table S1) and 998 DEGs in NV HSC (Table S2) when compared to wild-type HSC (DEGs were defined as a difference in the expression level over 1.5-fold, adjusted the *p* value < 0.05 (DESeq2 R package)). There were only 59 overlapping DEGs in NA10hd and NV (Figure 4A). GO enrichment analysis using the NA10hd or NV DEGs showed that these DEGs were largely related to pathways of the cell cycle, cell division, and DNA replication and nuclear division (Figure 4B,C). Only 14 of the 59 (<20%) overlapping DEGs from NA10hd and NV HSC showed a similar expression trend (Figure 4D). However, more than 80% of the overlapping DEGs had opposite expression trends in NA10hd and NV. There were approximately 17 (28%) DEGs that were up-regulated in NA10hd HSC, but down-regulated in NV HSC including *H19* and *Igf2* that regulate HSC quiescence [17] (Figure 4E). In addition, 28 (>50%) DEGs were up-regulated in NV HSC, but down-regulated in NA10hd HSC (Figure 4F).

**Figure 5:**
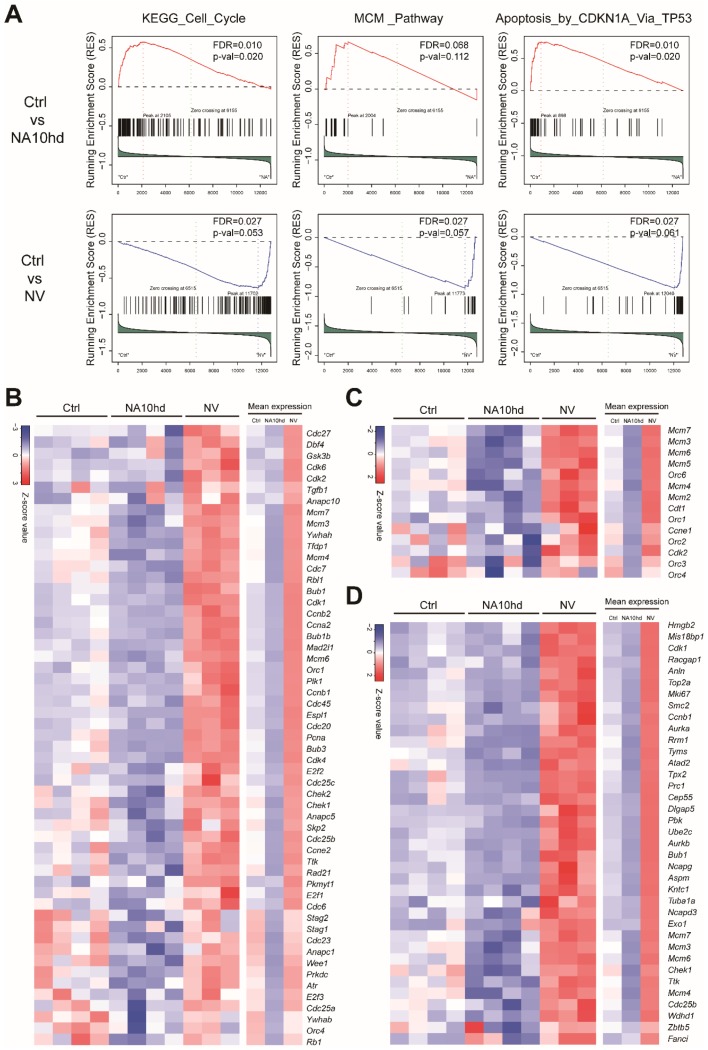
Gene set enrichment analysis (GSEA) showed opposite-signaling patterns in HSC altered by NA10hd and NV expression. (**A**) The gene sets of the KEGG_Cell_Cycle, MCM_Pathway, and Apoptosis_by_CDKN1A_Via_TP53 signaling pathways from the public GSEA database (http://software.broadinstitute.org/gsea/downloads.jsp) were used for GSEA analysis using the GSEA R package. Nominal *p* value, empirical phenotype-based permutation test (*p* < 0.05, FDR < 0.25). (**B**) Heatmaps represent the expression of leading-edge genes in a KEGG_Cell_Cycle pathway. (**C**) Heatmaps represent the expression of leading-edge genes in an MCM pathway. (**D**) Heatmaps represent the expression of leading-edge genes in Apoptosis_by_CDKN1A_Via_TP53 signaling pathways. Columns represent the indicated biological replicates of each population (**B**–**D**).

Further gene set enrichment analysis (GSEA) analysis showed that NA10hd and NV regulated the cell cycle, MCM_Pathway, and Apoptosis_by_CDKN1A_Via_TP53 signaling pathways in an opposite manner (Figure 5A). Furthermore, the leading-edge genes analysis showed that the cell cycle-related genes including the cyclin dependent kinase (Cdk) family genes (*Cdk1*, *Cdk2*, *Cdk4*, *Cdk6*) and cell division cycle (Cdc) family genes (*Cdc7*, *Cdc6*, *Cdc16*, *Cdc27*, *Cdc23*, *Cdc25a*, *Cdc25b*, *Cdc25c*) were upregulated in NV HSC but down-regulated in NA10hd HSC (Figure 5B). At the same time, the cell cycle and initiator proteins of DNA replication related genes mini-chromosome maintenance complex (Mcm) family genes (*Mcm2*, *Mcm3*, *Mcm4*, *Mcm5*, *Mcm6*, *Mcm7*) and Origin Recognition Complex (ORC) family genes (*Orc1*, *Orc2*, *Orc3*, *Orc4*, *Orc6*) (Figure 5C) had the same expression trend as Cdk or Cdc family genes. In addition, cell apoptosis related genes *Hmgb2* [18], *Ccnb1* [19], *Aurka* [20], *Rrm1* [21], *Tpx2* [22], *Prc1* [23], and *Pbk* [24] were upregulated in NV HSC while downregulated in NA10hd HSC (Figure 5D). Genes that regulate the hematopoietic stem cell activity [25,26,27,28] including *Hoxa10*, *Hoxa7*, *Hoxa9*, *Ski*, *Prdm16*, *Vps72*, *Hoxb4*, and *Akt1* were up-regulated in NA10hd HSC, while only *Trim27* and *Smarcc1* were up-regulated in NV HSC (Figure S3). Collectively, the above results demonstrate that NA10hd and NV conferred competitiveness on HSC by distinct mechanisms.

### 2.5. Single NAV-HSC Survive and Expand in Feeder-Free Medium and Its Derivatives Fully Repopulate Multi-Lineage Hematopoiesis in Recipients

**Figure 6:**
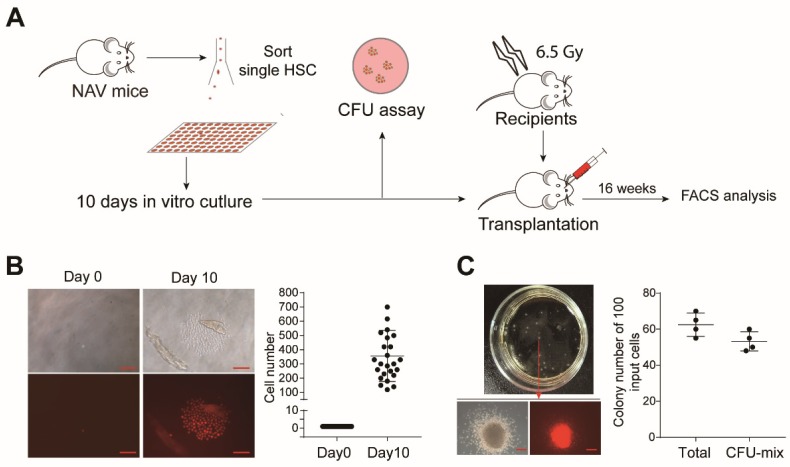
Evaluation of multi-lineage potential by a colony forming assay using 10-day-cultured single NAV HSC. (**A**) Schematic diagram of feeder-free culture of single NAV HSC and subsequent function assessment. (**B**) Colony morphology formed by single NAV HSC after the 10-day culture. Single HSC were sorted into individual 96-wells and cultured in defined feeder-free medium (StemSpan™ SFEM plus with 100 ng/mL mSCF 20 ng/mL human IL11, β-mercaptoethanol (2000-fold diluted)). Representative images from day 0 and day 10 were shown. The absolute cell number of cells within each colony was counted at day 10, and only colonies with more than 100 cells per well were further analyzed. Scale bars, 200 µm. (**C**) Colony-forming unit (CFU) assay of in vitro cultured cells. Half of the cells from individual colonies with homogeneous morphology were used as input for the CFU assay. Total CFU and CFU-mix colonies were counted. Scale bars, 200 µm.

HSC are susceptible to stress and prone to die in vitro in the absence of feeder cells, which can only be expanded with cell lines such as AFT024 and UG26 [29,30]. It is also reported that adult mouse HSC (E-SLAM cells) transduced with a retrovirus expressing NA10hd could expand efficiently during the 14-day culture with an addition of IL-3, IL-6, and SCF [4]. We wondered whether single NAV-HSC could overcome the culture stress and expand in a feeder-free condition. We sorted single Lin (CD2, CD3, CD4, CD8, B220, Gr1, Mac1, Ter119)^−^CD48^−^c-Kit^+^Sca1^+^CD135^−^CD150^+^ NAV-HSC or WT-HSC into individual wells of a 96-well low adhesion plate containing 150 µL culture medium (StemSpan™ SFEM plus with 100 ng/mL mSCF, 20 ng/mL human IL11, and β-mercaptoethanol (2000-fold diluted)). To evaluate the stemness preservation, colony-forming unit (CFU) and transplantation assay were performed using the single cell-derivatives after a 10-day culture (Figure 6A). Amazingly, more than 30% (25/80) of wells with single NAV-HSC input proliferated to produce colonies composed of more than 100 cells (Figure 6B). In contrast, we observed very rare daughter cells from the WT HSC group (data not shown). Notably, certain wells showed homogenous morphology with uniform offspring cells in size. We divided the recovered cells from each well into two equal aliquots, one for an in vitro colony-forming assay (CFU-C) and the other for in vivo transplantation. As expected, only the cells with homogeneous morphology daughter cells (Figure S4A) produced CFU-Mix colonies (Figure 6C), but not the wells with heterogeneous size derivatives (Figure S4B).

**Figure 7:**
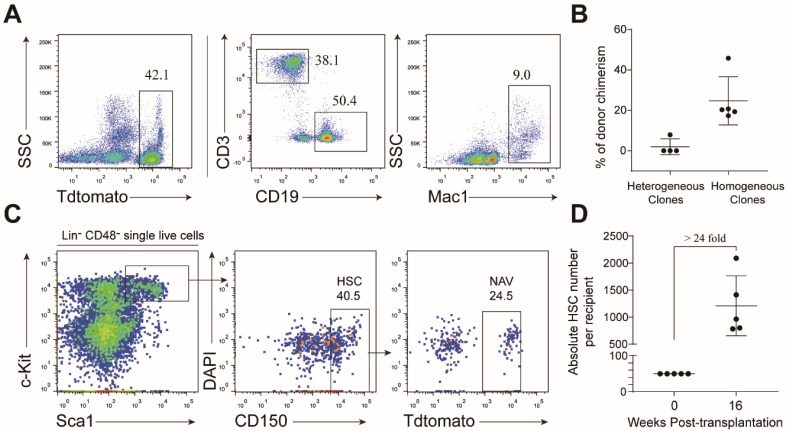
Evaluation of multi-lineage potential of cultured single NAV HSC by transplantation. (**A**) Representative flow cytometric analysis of donor (NAV)-derived lineages in recipients transplanted with 10-day cultured cells. Fifty cells collected from each colony were transplanted into sublethally irradiated (6.5 Gy) mice (CD45.1). Flow cytometric analysis was performed to assess the lineages of donor-derived cells in peripheral blood 16 weeks post-transplantation. (**B**) The percentage of donor-derived cells in peripheral from heterogeneous and hemogeneous clones. Data are presented as means ± SD. (**C**) Flow cytometric analysis of donor (NAV)-derived phenotypic HSC in the bone marrow of recipients 16 weeks post-transplantation. (**D**) The absolute number of donor (NAV)-derived HSC. Data are represented as means ± SD. Data are either representative plots from two independent experiments (**A**,**C**), or are pooled from two independent experiments (**B**,**D**).

To further evaluate stemness of single NAV HSC derivatives, in addition to the CFU-C assay, we transplanted 50 cells from representative individual wells into sub-lethally irradiated mice (CD45.1). Of the transplanted nine colonies, five were homogenous and four were heterogeneous. Cells from six individual wells, including five homogenous colonies and one heterogeneous colony, successfully reconstituted multi-lineage hematopoiesis over 16 weeks after transplantation (Figure 7A,B). To further confirm whether these mice contained donor-derived hematopoietic stem cells, we performed flow cytometric analysis of the bone marrow nucleated cells. As expected, phenotypic donor HSC were detected in the bone marrow of the recipients of homogenous colonies (Figure 7C), but not in the recipients transplanted with heterogeneous colonies (data not shown). The estimated absolute number of donor-derived phenotype HSC expanded around 24-fold than their input (Figure 7D). Collectively, our data indicated that NAV-modified HSC expanded in vitro and preserved their stemness in a feeder-free medium condition.

## 3. Materials and Methods

### 3.1. Mice

C57BL/6 (CD45.1) and Vav-Cre strain (CD45.2) mice were purchased from the Jackson Laboratory. C57BL/6 (CD45.2) were from the Beijing Vital River Laboratory Animal Technology Ltd. NA10hd^LSL/+^ mice were generated by targeting a mouse ES line (C57BL/6 line, Beijing Biocytogen Co., Ltd.) through homologous recombination at the ROSA26 locus. NA^LSL/+^ mice were subsequently bred with Vav-Cre mice to generate NA10hd^LSL/+^ and Vav-Cre (NA10hd) compound mice. Mice expressing the conditional oncogenic NrasG12D mutation (a gift from Dr. Jing Zhang lab at the University of Wisconsin-Madison, Wisconsin, USA) were crossed with Vav-Cre mice to generate LSL Nras^G12D/+^ and the Vav-Cre compound (NV) mice. Mice were housed in the SPF grade animal facility of the Guangzhou Institution of Biomedicine and Health, Chinese Academy of Science (GIBH, CAS, China). All animal experiments were approved by the Institutional Animal Care and Use Committee of Guangzhou Institutes of Biomedicine and Health (IACUC-GIBH).

### 3.2. Competitive Transplantation

All the competitive transplantation of total bone marrow were performed at the ratio of 1:1 (0.25 million to 0.25 million). The recipients (CD45.1) of all competitive transplantation were lethal irradiated (2 × 5.0 Gy). The donor-derived chimeric ratios of peripheral blood cells were analyzed regularly and the HSC compartment was analyzed 20 weeks post-transplantation. Donor-derived cells from NA10hd and NAV were identified with Tdtomato.

### 3.3. Flow Cytometric Analysis

Antibodies for the hematopoietic lineage and hematopoietic progenitors or stem cells analysis: CD45.2 (104), CD2 (RM2-5), CD3e (145-2C11), CD4 (RM4-5), CD8a (53-6.7), Ter119 (TER-119), Mac1 (M1/70), B220 (6B2), Gr1 (RB6-8C5), CD19 (1D3/CD19), CD90.2 (53-2.1), CD48 (HM48-1), IL-7R (A7R34), Sca1 (E13-161.7), c-Kit (2B8), CD34 (RAM34), and CD16/32 (93) antibodies were purchased from eBiosciences, and CD150 (TC15-12F12.2) was purchased from Biolegend. DAPI (#D9542-50MG) was purchased from Sigma.

All the nucleated cells harvested from moue tissues were first treated with ACK solution, and then blocked with the CD16/32 antibody. For lineages staining, blocked nucleated cells were incubated with antibodies mixture of CD19-FITC, Mac1-PE-Cy7, and Thy1.2-APC. For myeloid progenitors staining, bone marrow cells were stained with antibodies mixtures of Lin (CD2, CD3e, CD4, CD8a, Ter119, Mac1, B220, Gr1)-FITC, Sca1-PerCP-Cy5.5, c-Kit-APC-eFluor^®^ 780, CD34-Alex700, and CD16/32-PE-Cy7. For lymphocyte progenitors staining, BM cells were stained with the antibodies mixture of Lin (CD2, CD3e, CD4, CD8a, Ter119, Mac1, B220, Gr1)-FITC, IL-7R-APC, Sca1-PerCP-Cy5.5, c-Kit-APC-eFluor^®^ 780. For hematopoietic stem cell (HSC) and multipotent progenitor (MPP) staining, BM cells were stained with the antibodies mixture of Lin (CD2, CD3e, CD4, CD8a, Ter119, Mac1, B220, Gr1)-FITC, CD48-FITC, Sca1-PerCP-Cyanine5.5, c-Kit-APC-eFluor^®^ 780, CD45.2-PE (only in NV and CD45.1 competitive transplantation recipients), and CD150-PE-Cy7. Lastly, all the cells were resuspended in DAPI solution.

The stained cells were analyzed on LSR Fortessa (BD Bioscience), and the data were analyzed using Flowjo software (FlowJo).

### 3.4. Single HSC Feeder-Free Culture

Individual NAV or wild-type (WT) HSC was sorted into a 96-well low adhesion plate containing 150 µL culture medium (StemSpan™ SFEM (stem cell, #09650) and 100 ng/mL mSCF + 20 ng/mL human IL11 + β-mercaptoethanol (2000 fold diluted)). A half-medium change was performed every other day. Colonies with more than 100 cells in each well were divided into equal aliquots. One aliquot of around 50 cells were plated for CFU-C colony-forming assay following the manufacturer’s instructions (STEMCELL Technologies) and the other aliquot of 50 cells were transplanted into sub-lethally irradiated (6.5 Gy) recipients (CD45.1) after a 10-day in-vitro culture.

### 3.5. RNA-Seq and Data Analysis

NA10hd, NV, and littermate control (Ctrl) HSC were sorted separately into 200 µL DPBS-BSA buffer (0.5% BSA) using a 1.5 mL Eppendorf tube. The cDNA of sorted 1000-cell aliquots were generated and amplified, as described previously [31]. Then qualities of the amplified cDNA were examined by qPCR analysis of housekeeping genes (B2m, Actb, Gapdh). Samples that passed quality control were used for subsequent sequencing library preparation by illumina Nextera XT DNA Sample Preparation Kit (FC-131-1096). All libraries were sequenced by illumina sequencer NextSeq500 (illumina). The fastq raw data files were generated using illumina bcl2fastq software and uploaded to the Gene Expression Omnibus public database (GSE118809). All the fastq files were aligned to the NCBIM37 mouse genome by Hisat2 software. Normalization of gene expression and differential expression gene analysis were performed by DESeq2. Heatmaps were plotted using gplots (heatmap.2). Gene set enrichment analysis (GSEA) was performed as described [32].

### 3.6. Statistical Analysis

The data were represented as mean ± SD. Two-tailed independent Student’s t-tests were performed for a comparison of two groups of data (SPSS v.23, IBM Corp., Armonk, NY, USA). P values of less than 0.05 were considered statistically significant (* *p* < 0.05, ** *p* < 0.01, *** *p* < 0.001).

### 3.7. Data Availability

The data that support the findings of this study are available from the corresponding author upon request. All RNA-Seq data are in the GEO database with accession code GSE118809.

## 4. Discussion

NA10hd transgenic mice had normal multi-lineage hematopoiesis, demonstrated by normal lineage and progenitor contribution indistinguishable from control mice. A previous study reported that knockdown of *Pbx1* improves the competitiveness of HoxB4-overexpressing HSC and that the capacity of NUP98-HoxA10HD to induce HSC expansion does not require a PBX-interacting motif [8,33]. Consistent with these findings, our competitive transplantation assay further confirmed that NA10hd with only the homeodomain of *Hoxa10* in the fusion improved the competitiveness of hematopoietic stem cells. This competitive advantage may be due to dominance of NA10hd HSC in the HSC compartment. Moreover, recipient mice with NA10hd HSC transplantation did not develop myeloproliferative disease until the end of the experiment. This is consistent with a previous study, which shows restriction of NUP98-fusion to the homeodomain of Hoxa10 blunts lineage bias and leukemogenic potential of Nup98-Hoxa10 [8].

It was previously reported that NrasG12D could sustainably increase the competitiveness of HSC [14,15]. It is of particular interest to show that NV HSC had stronger competitiveness than NA10hd HSC. The transcriptomic analysis of NA10hd HSC and NV HSC showed that both NV and NA10hd affected the signals of DNA replication, the cell cycle, and apoptosis of HSC. However, NA10hd HSC and NV HSC represent an opposite trend in regulating the cell cycle, DNA replication, and Apoptosis_by_CDKN1A_Via_TP53 signaling pathways. NA10hd may enhance the transplantation and proliferative capacity of hematopoietic stem cells through HSC activators such as Hoxb4, Hoxa9, Hoxa10, Prdm16, Ski, and Vps72 etc. [25,34,35,36], and maintain the stemness of hematopoietic stem cells through an H19-Igf2-related pathway [17]. In contrast, NV may enhance the engraftment advantage of hematopoietic stem cells by regulating the Stat5 signaling pathway and MEK/ERK pathway [11,14,15,37]. Of note, we have identified a signature of 14 genes, which were commonly downregulated or upregulated by both NA10hd and NrasG12D HSC (Figure 4D). Functional analysis of HSC with these 14 signature genes would shed light on the mechanism of increased competitiveness of HSC in both NA10hd and NrasG12D HSC. *Flt3l*, which is one of these signature genes, has been reported to regulate multipotent stem and progenitor cells and enhance their proliferation [38].

The co-expression of NA10hd and Nras in hematopoietic stem cells conferred the strongest engraftment advantage in vivo than NV or NA10hd alone. In addition, a transplantation assay of single NAV HSC-derived cells after 10-day feeder-free in vitro culture showed that the NAV HSC derivatives could efficiently reconstitute the whole blood lineages in all the recipients. These results suggested that NA10hd and NrasG12D gene could synergistically enhance the competitiveness and preserve stemness of hematopoietic stem cells. Since NA10hd and NV HSC employs mostly distinct signaling pathways yet share a similar trend for a group of genes to confer competitiveness, how NA10hd and NV collaborate to eventually confer a competitive advantage in HSC deserves further investigation. In addition, it would be of great interest to investigate whether the NAV modification could be introduced into pluripotent stem cells to capture bona fide HSC regenerated from pluripotent stem cells.

In conclusion, HSC with NrasG12D and NUP98-HOXA10HD gene-modifications possess a super engraftment advantage and preserved their multi-lineage potential after in vitro culture. Thus, we established a super-sensitive readout system reporting the existence of HSC on the culture condition at single cell resolution, which could be beneficial for screening induced HSC from pluripotent stem cells.

## Supplementary Materials

The following are available online at https://www.mdpi.com/2073-4409/8/9/951/s1. Figure S1. Flow cytometric analysis of multi-lineage hematopoiesis and hematopoietic progenitors in aged NA10hd transgenic mice. Figure S2. Sorting strategy of HSC. Figure S3. Heatmaps present the relative expression level of genes that affect hematopoietic stem cell activity. Figure S4. Colony morphology formed by single NAV-HSC after 10-day culture on feeder-free condition. Table S1. Differential expression genes between NA10hd-HSC and control HSC. Table S2. Differential expression genes between NV-HSC and control HSC.
