# Supplementary material for: Synergy of NUP98-HOXA10 Fusion Gene and NrasG12D Mutation Preserves the Stemness of Hematopoietic Stem Cells on Culture Condition
Source: Cells. 2019 Aug 22;8(9):951. doi: 10.3390/cells8090951 (PMC6770072; doi:10.3390/cells8090951)
Supplement: Supplementary file 1 [file cells-08-00951-s001.zip › Supplementary Material/Supplementary Figure legends.docx]

**Supplementary Figure legends**

**Figure S1. Flow cytometric analysis of multi-lineage hematopoiesis and hematopoietic progenitors in aged NA10hd transgenic mice.** Bone marrow nucleated cells of 12-month-old NA10hd^LSL/+^;Vav-Cre mice or littermate control (Ctrl) mice (NA^LSL/+)^ were analyzed. (A-D) Flow cytometric analysis of hematopoietic lineages in peripheral blood and bone marrow. Representative flow plots of hematopoietic lineage analysis in peripheral blood (A), hematopoietic stem/progenitor cell (HSC/MPP) (B), common lymphoid progenitor (CLP) (C) and myeloid progenitor (MP) (D) in bone marrow of NA10hd and control mice are shown. Data are representative of two independent experiments. (E) The absolute cell numbers of MPPs, Lin^-^CD48^–^c-Kit^+^Sca1^+^CD135^-^ CD150^–^ ST-HSC and Lin^–^CD48^–^c-Kit^+^Sca1^+^CD135^-^CD150^+^ LT-HSC in one million bone marrow cells of NA and control mice were calculated. Lin cocktail includes CD2, CD3, CD4, CD8, B220, Mac1, Gr1, Ter119. (F) The percentages of Mac1^+^ myeloid cells and CD19^+^ B cells and CD90.2^+^ T cells in peripheral blood of NA10hd mice or control mice. The absolute number of Lin^–^ CD127^+^c-Kit^mid^ Sca1^+^ CLPs (G), Lin^–^CD127^–^c-Kit^+^Sca1^+^CD16/32^+^CD34^+^ GMPs, Lin^–^CD127^–^c-Kit^+^Sca1^+^CD16/32^mid^CD34^mid^ CMPs, and Lin^–^CD127^–^c-Kit^+^Sca1^+^ CD16/32^-^CD34^-^ MEPs (H) in one million bone marrow cells of NA and control mice were calculated based on respective percentage measured by flow cytometry analysis. Data are represented as means ± SD. Unpaired Student’s t-test (two-tailed) was performed. N = 3 mice, * p <0.05, * * p <0.01, * * * p <0.001, ns indicates not significant.

**Figure S2. Sorting strategy of HSC.** Bone marrow nucleated cells were first incubated with purified CD16/32 antibodies for ten minutes on ice, and then stained with the antibodies mixture of Lin (CD2, CD3, CD4, CD8, CD19, B220, Gr1, Mac1, Ter119), CD48, c-Kit, Sca1, CD135 and CD150. The HSC were sorted by Lin/CD48^-^ c-Kit^-^ Sca1^-^ CD135^-^ CD150^+^.

**Figure S3. Heatmaps present the relative expression level of genes that affect hematopoietic stem cell activity.** The hematopoietic stem cell activity related genes were collected from literatures [1-4]. Heatmaps were plotted by gplots (heatmap.2) R package. Columns represent the indicated biological replicates of each population.

**Figure S4. Colony morphology formed by single NAV-HSC after 10-day culture on feeder-free condition.** (A) Representative images of the colonies with homogeneous daughter cells. (B) Representative images of the colonies with heterogeneous daughter cells. Single NAV-HSC were sorted into 96-well low adhesion U plate containing with 150 µL culture medium (150 µL culture medium (StemSpan™ SFEM (stem cell, #09650) 100 ng/mL mSCF + 20 ng/mL human IL11 + β-mercaptoethanol (1:2000)). Half-medium replacement was performed every other day. Scale bars, 200 µm.

**References**

1. Deneault E, Cellot S, Faubert A, Laverdure JP, Frechette M, Chagraoui J, et al. A functional screen to identify novel effectors of hematopoietic stem cell activity. Cell. 2009;137(2):369-79. Epub 2009/04/22. doi: 10.1016/j.cell.2009.03.026. PubMed PMID: 19379700.

2. Lawrence HJ, Christensen J, Fong S, Hu YL, Weissman I, Sauvageau G, et al. Loss of expression of the Hoxa-9 homeobox gene impairs the proliferation and repopulating ability of hematopoietic stem cells. Blood. 2005;106(12):3988-94. Epub 2005/08/11. doi: 10.1182/blood-2005-05-2003. PubMed PMID: 16091451; PubMed Central PMCID: PMC1895111.

3. Brun AC, Bjornsson JM, Magnusson M, Larsson N, Leveen P, Ehinger M, et al. Hoxb4-deficient mice undergo normal hematopoietic development but exhibit a mild proliferation defect in hematopoietic stem cells. Blood. 2004;103(11):4126-33. Epub 2004/02/14. doi: 10.1182/blood-2003-10-3557. PubMed PMID: 14962901.

4. Ferrell CM, Dorsam ST, Ohta H, Humphries RK, Derynck MK, Haqq C, et al. Activation of stem-cell specific genes by HOXA9 and HOXA10 homeodomain proteins in CD34+ human cord blood cells. Stem Cells. 2005;23(5):644-55. Epub 2005/04/26. doi: 10.1634/stemcells.2004-0198. PubMed PMID: 15849172.
