# Supplementary figures and images for: Synergy of NUP98-HOXA10 Fusion Gene and NrasG12D Mutation Preserves the Stemness of Hematopoietic Stem Cells on Culture Condition
Source: Cells. 2019 Aug 22;8(9):951. doi: 10.3390/cells8090951 (PMC6770072; doi:10.3390/cells8090951)

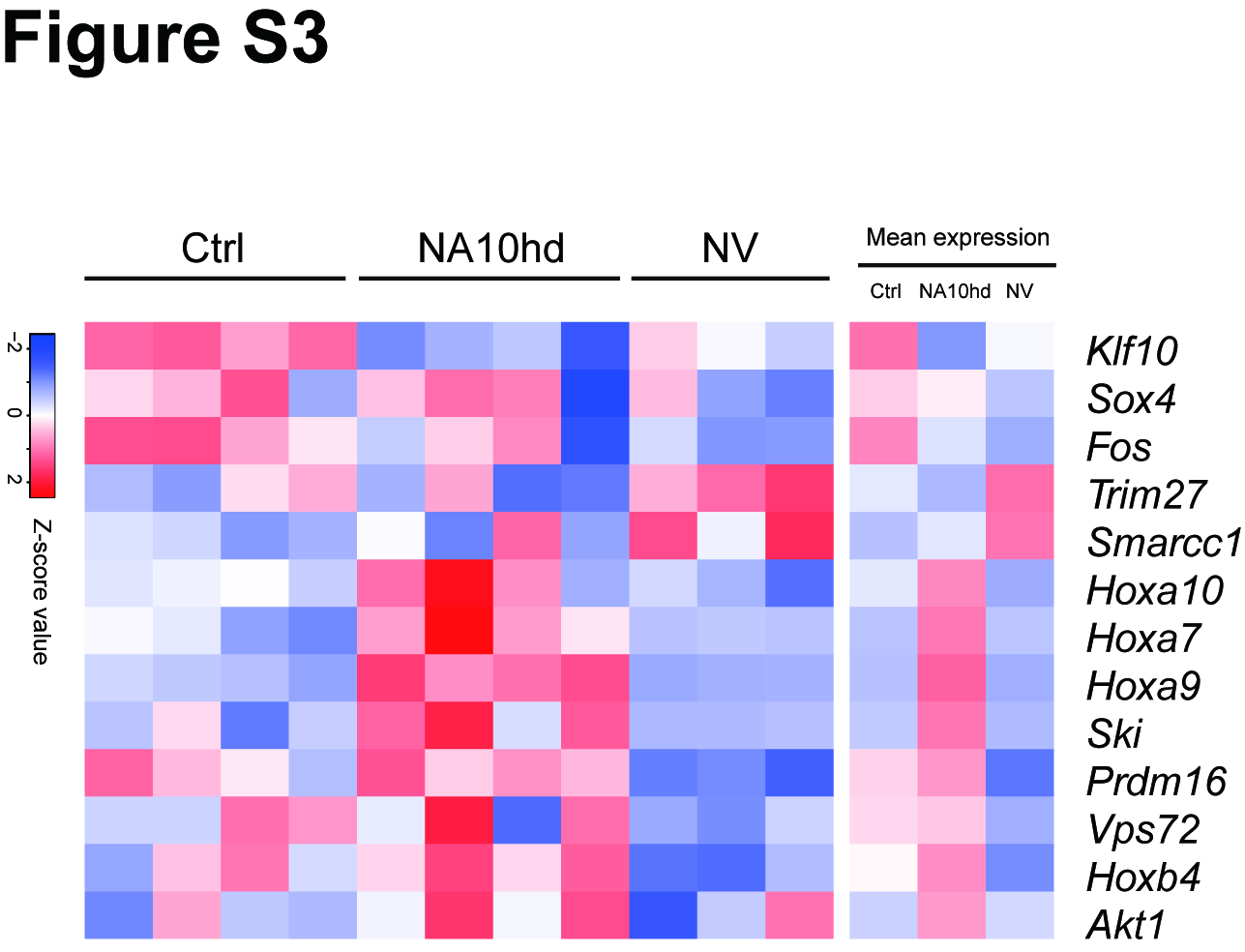

Supplement: Supplementary file 1 [file cells-08-00951-s001.zip › Supplementary Material/Figure S3 Stemness_genes.tif]

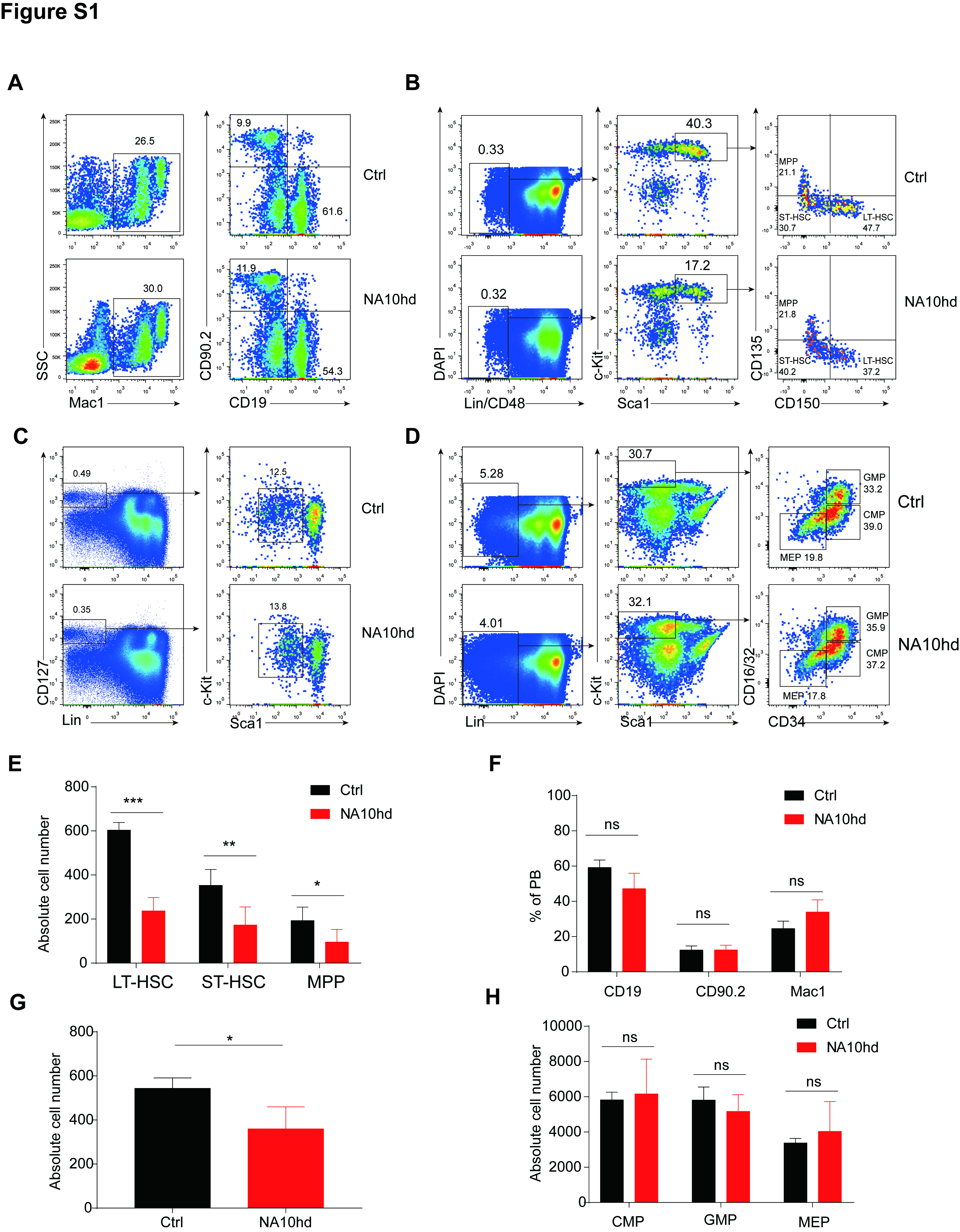

Supplement: Supplementary file 1 [file cells-08-00951-s001.zip › Supplementary Material/Figure S1 Aging mice.tif]

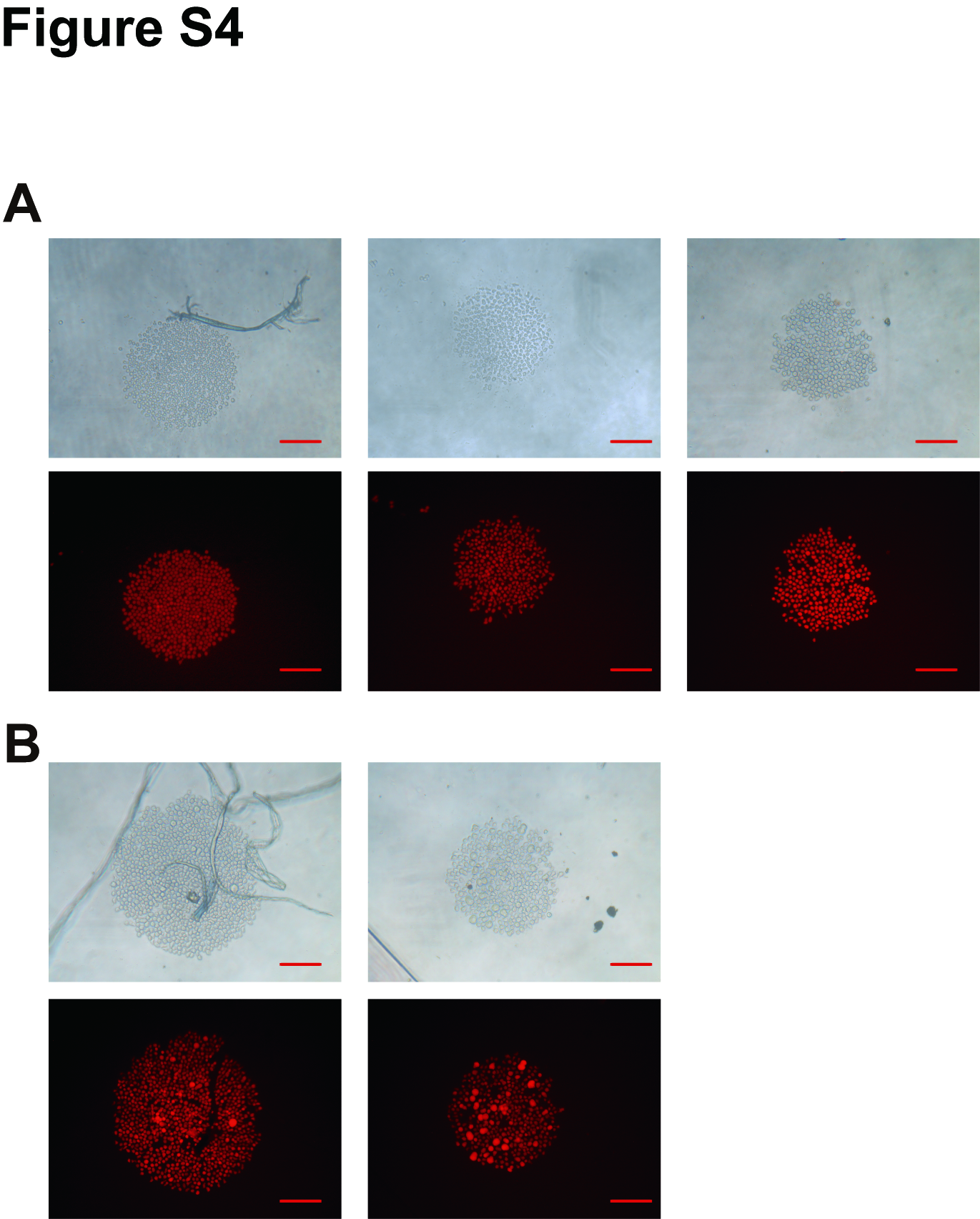

Supplement: Supplementary file 1 [file cells-08-00951-s001.zip › Supplementary Material/Figure S4.tif]

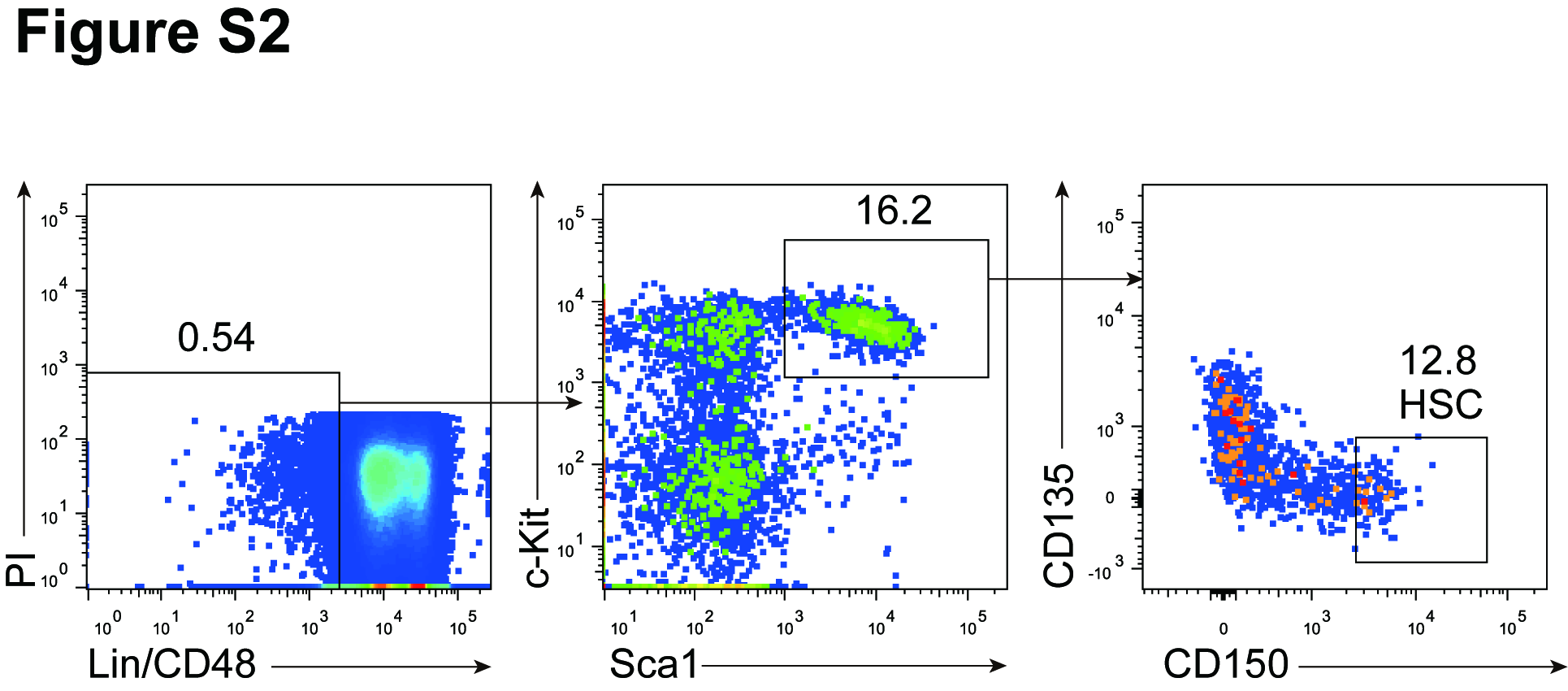

Supplement: Supplementary file 1 [file cells-08-00951-s001.zip › Supplementary Material/Figure S2 HSC_sorting.tif]
